# Nutritional supplements to improve esports player performance: a systematic review of randomized controlled trials

**DOI:** 10.1080/15502783.2025.2603303

**Published:** 2025-12-22

**Authors:** Da Huang, Yan Zheng, Ming Xu, Wenying Huang

**Affiliations:** a College of Physical Education, Jiangxi University of Technology, Nanchang, People's Republic of China; b College of Physical Education, Jiangxi Normal University, Nanchang, People's Republic of China

**Keywords:** Esports, nutritional supplements, caffeine, cognitive function, gaming performance, randomized controlled trial

## Abstract

**Background:**

Esports has become a globally popular competitive activity. The performance of esports athletes depends not only on daily skill training but also on cognitive function, reaction speed, and psychological mood. In recent years, nutritional supplements have attracted widespread attention as a potential adjunctive treatment. However, their actual effects lack systematic evaluation. Objective: The primary aim of this study is to comprehensively review existing evidence and assess the impact of nutritional supplements on the performance of esports athletes, including cognitive, psychological, and gaming aspects.

**Methods:**

As of June 17, 2025, randomized controlled trials (RCTs) or randomized crossover trials investigating the effects of nutritional supplements on cognitive function, psychological mood, and competitive performance in esports players were retrieved from the PubMed, Scopus, Web of Science, and Embase databases. Two researchers independently extracted key information and data from the literature. The methodological quality of the included studies was assessed using the Physical Therapy Evidence Database (PEDro) scale.

**Results:**

A total of 13 randomized controlled trials were included, comprising 10 randomized crossover trials and 3 randomized controlled trials. The study population comprised 466 participants. The methodological quality of the studies, assessed by the PEDro scale (score range 6-10), was good to excellent. The studies included 18 nutritional supplement protocols, with 14 protocols involving pure caffeine or caffeine-containing supplements. Other protocols included active substances such as inositol-enhanced arginine silicate (ASI + I), *γ*-aminobutyric acid (GABA), and microalgae extracts. Based on existing evidence, some nutritional supplements are associated with three aspects of competitive performance among esports players: 1) Esports players demonstrate significant improvements in attention and executive function, which are closely related to gaming. 2) Improvements in esports players' psychological mood are manifested as increased vitality and reduced fatigue/negative emotions. 3) Improvements in gaming performance are primarily focused on shooting performance, such as increased shooting scores and accuracy, as well as reduced reaction times.

**Conclusion:**

Specific nutritional supplements may improve esports players' cognitive function, psychological mood, and gaming performance.However, these findings represent preliminary evidence based on the heterogeneity of the included studies and raise concerns regarding the overall risk of bias in over half of the research. Furthermore, the small sample sizes and focus on amateur players limit the generalizability of the results. Consequently, caution is warranted when interpreting these findings.Future clinical studies are needed to standardize supplementation protocols, dosage, and measurement methods to confirm the benefits of nutritional supplements for esports players.

## 1. Introduction

Electronic sports (esports) are defined as ‘organised competitive digital games that vary in terms of their level of professionalism’ [1]. As an emerging competitive domain, esports has experienced rapid global growth, with total viewership reaching 532 million in 2023. The market generated $1.38 billion in revenue, primarily driven by sponsorships (60%). Projections indicate growth to $2.15 billion by 2026, with the Asia-Pacific region dominating current revenues (53%) [2]. With the International Olympic Committee (IOC) including esports as an official Olympic event and the growing number of national competitions, both participation and industry scale are expected to continue expanding.

Unlike traditional sports, esports performance relies more on neurocognitive functions than physical strength and endurance. Esports places extremely high demands on players' cognitive abilities. Professional players typically need to possess high-intensity information processing capabilities (including reaction speed and dynamic visual processing); deep strategic and computational abilities (including resource and probability calculations, tactical simulation, and prediction); and cognitive flexibility and interference resistance (rapid cognitive switching, stress resistance, and sustained focus) [3,4]. In summary, esports players must demonstrate strong abilities across various cognitive dimensions, such as attention (selective, sustained, and divided attention), executive function (inhibition control, cognitive flexibility, and working memory), processing speed (simple reaction and complex processing), and memory (visual-spatial memory). Research indicates that players exhibit superior cognitive function compared to non-players [5–7].

Esports competitions involve prolonged and intense screen exposure, which poses a significant challenge to esports players' cognitive health and emotional well-being. Research has found that prolonged exposure to blue light affects athletes' sleep quality, athletic performance, and cognitive function [8]. Excessive esports training and competition can lead to symptoms such as fatigue, burnout, eye discomfort, and difficulty concentrating [9]. Among esports players, nutritional supplements [10], sleep regulation [11], and physical exercise [12] have been identified as factors associated with cognitive function and psychological mood.

For instance, sleep regulation has been found to effectively improve cognitive function in physically active young populations [13]. In studies targeting esports players, sleep deprivation has been shown to adversely affect competition performance [11]. Nutritional supplements are considered a key factor in enhancing athletic performance, including for esports players. Existing research indicates that nutritional interventions, such as caffeine, can effectively improve athletes' competitive and cognitive performance [14,15]. In the esports field, the use of nutritional supplements to enhance players' cognitive and gaming performance has become a research hotspot in the fields of sports and nutrition [10,16,17]. To maintain a high competitive level during competitions and training, players often consume caffeinated beverages or other energy drinks containing natural ingredients [18–20].

A previous systematic review synthesised the effects of dietary supplements on cognitive function in players but did not provide detailed reporting on adverse events related to gaming performance or psychological states. Furthermore, no relevant studies have specifically synthesised RCTs on nutritional supplements in esports. Therefore, this study integrates evidence from RCTs to evaluate the effects of nutritional supplements on esports player of varying skill levels, focusing on safety and efficacy. Safety encompasses adverse events and side effects, while efficacy covers cognitive function, gaming performance, and psychological mood. This may provide preliminary evidence for nutritional practices in esports and clarify future research directions.

## 2. Methods

### 2.1. Protocol registration

This study followed the Preferred Reporting Items for Systematic Reviews and Meta- Analyses (PRISMA) guidelines [21,22] (see the PRISMA checklist in Supplementary Material Table 1). This study has been registered in the International Prospective Register of Systematic Reviews (PROSPERO) (CRD420251105012).

### 2.2. Search strategy

RCTs, or randomised crossover trials, investigating the effects of nutritional supplements on cognitive function and gaming performance in esports players were retrieved from the PubMed, Scopus, Web of Science, and Embase databases. Search terms and Boolean operators: “esports” OR “e-sports” OR “game” OR “gamer” OR “video gaming” AND “nutrition” OR “supplementation” OR “placebo” OR “caffeine” OR “drink” OR “capsule” OR “supplement” AND “randomised controlled trial” OR “randomised crossover trial.” The search was conducted up to 17 June 2025. Detailed search criteria are provided in Supplementary Table 2. To minimise the risk of study exclusion, a secondary search of the references of included studies was conducted (Table 1).

**Table 1.** PICOS framework included in the systematic review.

PICOS	Description
Population	Esports players (aged ≥ 18), excluding minors.
Intervention	Nutritional supplements (including caffeine, energy drinks, arginine silicate, etc.).
Comparetor	Placebo supplementation
Outcome	Cognitive performance, gaming performance, Psychological mood
Study design	Randomised controlled trial, randomised crossover trial

### 2.3. Inclusion and exclusion criteria

The PICOS framework was used to determine inclusion criteria (population, intervention, control group, outcome measures, and study design). The framework was followed. Population: Esports players or game participants aged 18 and above, excluding minors. Intervention: The intervention group consumed caffeine, energy drinks, arginine silicate, and other nutrients. Control group: The control group consumed a placebo with the same colour and packaging as the intervention group. Outcome measures: cognitive function outcomes, such as attention, executive function, reaction time, and alertness; psychological mood outcomes, such as fatigue, vitality, friendliness, and overall emotional distress; and gaming performance outcomes, such as game scores, time to complete game operations, shooting scores, and accuracy rates. Study design: Original peer-reviewed studies published in English-language journals, specifically single-blind, double-blind RCTs, or randomised crossover trials. Detailed information is provided in Table 2.

**Table 2.** Characteristics of the studies included in this systematic review.

Study/year	Country/Region	Sample size (M/F)	Level/Age	Height(cm)	Weight(kg)	BMI(kg/m^2^)	Supplementation protocol	Composition and dosage	Study design
Thomas et al. [24]	USA	*N* = 9 (9/0)	Elite /21 ± 2	177 ± 7	80.13 ± 13.18	25.60 ± 3.44	SG: Reload ^TM^ energy drink CG: Placebo	Caffeine 150 mg of L-theanine, phosphatidylserine, etc. 118 ml per serving (1.9 ± 0.3 mg/kg caffeine).	RCT, DB, PC, crossover, Washout period: 7 day
Tartar et al. [25]	USA	*N* = 60 (50/10)	Amateur/ SG: 27.8 ± 5.51 CG:29.3 ± 5.29	SG: 178.6 ± 6.67 CG:177.8 ± 9.41	SG:86.02 ± 17.27 CG:87.72 ± 15.64	SG:26.87 ± 4.74 CG:27.72 ± 4.38	SG: ASI + I CG: Placebo	Arginine silicate 1500 mg + Inositol 100 mg.	RCT, DB, PC, prospective.
Sainz et al. [26]	Spain	*N* = 15 (15/0)	Elite/ 22 ± 3	NR	NR	NR	SG: Caffeine. CG: Placebo	3mg/kg^–1^ . Time of intake: 45 minutes before testing.	RCT, DB, cross-over. Washout period: 3 day
Sowinski et al. [27]	USA	*N* = 26 (18/8)	Amateur/ 23.1 ± 5	171 ± 11	71.1 ± 13.8	20.7 ± 3.5	SG: ASI + I CG: Placebo	1600 mg Inositol Stabilised Arginine Silicate (containing 100 mg inositol).	RCT, DB, PC, crossover. Washout period: 7-14day.
Tartar et al. [28]	USA	*N* = 50 (50/0)	Amateur/ 20.52 ± 2.03	177.63 ± 9.11	81.67 ± 17.18	NR	SG1: CDT SG2: Caffeine CG: Placebo	Caffeine (125 mg) + TeaCrine ® (50 mg) + Dynamine® (75 mg), Caffeine (125 mg)	RCT, DB, crossover.
Bloomer et al. [29]	USA	*N* = 49 (47/2)	Amateur/ 22 ± 3	178 ± 8	82 ± 19	26 ± 5	SG1: AmaTea® SG2: Caffeine CG: Placebo	SG1: AmaTea® max: 270 mg caffeine + chlorogenic acid antioxidant SG2: pure caffeine 270 mg.	RCT, DB, PC, three-armed crossover. Washout period: 7 day
Leonard et al. [30]	USA	*N* = 61 (51/10)	Amateur/ 21.7 ± 4.1	173.4 ± 8.2	73 ± 13	24.2 ± 3.6	Microalgae extract containing fucoxanthin combined with guarana SG1: Low Dose SG2: High Dose CG: Placebo	Low Dose: 440 mg PT microalgae extract (containing 1% fucoxanthin) + 500 mg guarana (containing 40-44 mg caffeine). High Dose: 880 mg PT microalgae extract + 500 mg guarana.	RCT, DB, PC, parallel-arm.
Evans et al. [31]	USA	*N* = 49 (49/0)	Amateur/ 24.4 ± 4.5	NR	NR	NR	SG1: CDT SG2: Caffeine CG: Placebo	SG1: Caffeine (200 mg), TeaCrine (10 mg), Dynamine(50 mg) SG2: Caffeine (200 mg).	RCT, DB, PC, crossover, Washout period:7 day.
Wu et al. [32]	Taiwan	*N* = 9 (9/0)	Elite/20.8 ± 0.9	172.3 ± 1.2	72.8 ± 8.3	NR	SG: Caffeine CG: Placebo	3 mg/kg^–1^	RCT, SB, crossover. Washout period: 7 day
Rogers et al. [33]	Australia	*N* = 24 (22/2)	Amateur/ 22.29 ± 2.91	NR	83.4 ± 19.8	26.0 ± 6.2	Caffeine SG1: Low dose SG2: High dose CG: Plain water	1 mg/kg^–1^ of anhydrous caffeine consumed with 250 ml of water. 3 mg/kg^–1^ of anhydrous caffeine consumed with 250 ml of water.	RCT, SB, Washout period: 2-4 day
Jeyakodi et al. [34]	India	*N* = 60 (30/30)	Amateur/ SG:35.06 ± 5.76 CG:35.73 ± 5.71	SG:164.33 ± 5.53 CG:163.73 ± 4.57	SG: 60.24 ± 4.73 CG: 60.32 ± 4.33	NR	SG: Stadice^®^ CG: Placebo.	Mangiferin, 300mg/day, 7 day.	RCT, DB, PC
Schwager et al. [35]	USA	*N* = 45 (37/8)	Amateur/ 25.2 ± 5.8	173.23 ± 8.89	76.7 ± 15.4	25.5 ± 4.4	SG: C4S energy drink. CG: Placebo	Caffeine (200 mg); Citicoline, *N*-acetyl tyrosine, vitamin B3, vitamin B12 (dosage not specified).	RCT, DB, PC, crossover. Washout period: 7 day
Hara et al. [36]	Japan	*N* = 9 (9/0)	Amateur/ 21.33 ± 1.22	168.71 ± 7.38	60.89 ± 13.63	NR	SG: Oral GABA^®^ CG: Placebo	200 mg GABA® dissolved in 30 ml of water.	RCT, DB, crossover. Washout period: 7 day

Abbreviation: M: male, F: fame, BMI: body mass index, SG: supplementary group, CG: control group, RCT: randomised controlled trials, DB: double-blind, PC: placebo-controlled, SB: Single-blind, NR: not reported, ASI +I: arginine silicate+inositol, CDT:caffeine+ dynamine+ teaCrine, GABA: γ-aminobutyric acid.

Exclusion criteria: 1) Literature published in the form of papers, letters, reviews, conference abstracts, or commentaries. 2) Literature not written in English. 3) Studies that are not randomised crossover trials or RCTs.

### 2.4. Information extraction

The data obtained include 1) basic literature information (e.g. first author, year of publication, country/region of origin); 2) basic participant information (e.g. sample size, age, gender, height, weight, blood pressure, heart rate, etc.); 3) methodological quality information; 4) supplementary protocols (composition, dosage, and timing of administration of nutritional supplements, etc.); and 5) changes and comparisons between the supplement group and placebo group in relevant outcome measures, along with key findings. This extraction process was conducted independently by two authors (DH and YZ). When discrepancies arose, they were discussed with a third author (WYH) until consensus was reached.

### 2.5. Methodological quality and risk of bias assessment

The Physical Therapy Evidence Database (PEDro) scale [23] was independently used by two authors (MX and YZ) to assess the methodological quality of the included studies. When they could not reach an agreement, they discussed the matter with a third author (WYH) until a consensus was reached. Each of the 11 assessment items in the PEDro scale is scored on a scale of 0 to 1. A score of 1 indicates compliance with the criteria, while a score of 0 indicates non-compliance. Since item 1 is not scored, the maximum possible score for the remaining 10 items (items 2–11) is 10. Studies with a methodological quality score of ≥ 9 were regarded as excellent-quality research, those with a score of 6–8 as good-quality research, those with a score of 4–5 as fair-quality research, and those with a score of <4 as very poor-quality research.

Additionally, we employed the Cochrane Risk of Bias Assessment Tool (ROB 2.0) to evaluate the risk of bias across the literature. This tool encompasses five assessment domains: randomisation process bias; bias due to deviations from intended interventions; bias due to missing outcome data; bias in measurement of the outcome; and bias in selection of the reported result. Two researchers (MX and YZ) conducted independent assessments for each signal issue. In cases of disagreement, they consulted with the third author until a consensus was reached. All risk of bias assessments included three options: high risk, low risk, and some concerns.

## 3. Results

### 3.1. Literature screening process

**Figure 1.**
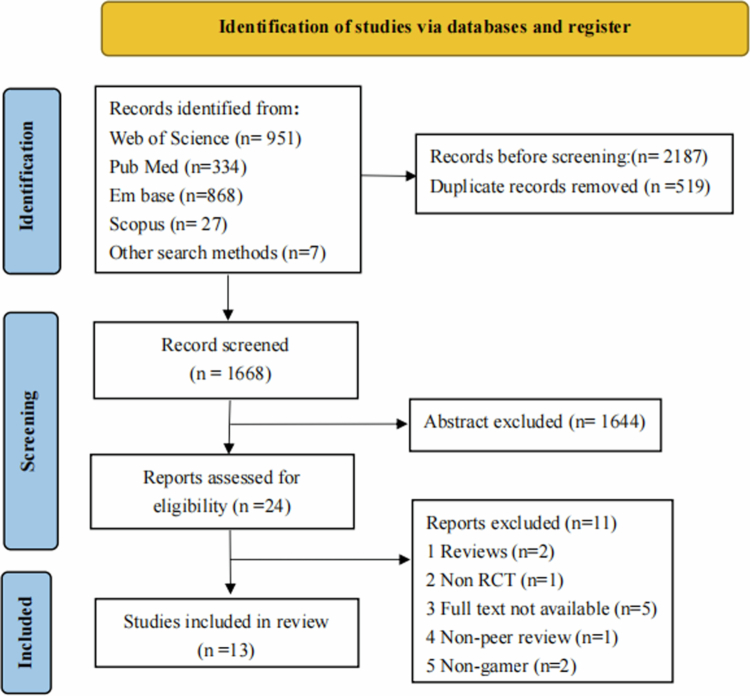
PRISMA flow diagram.

A total of 2,180 potential articles were retrieved from four electronic databases, and seven additional articles were obtained through other means. Using EndNote X9 software, 519 duplicate articles were removed. After reading the titles and abstracts, 1,644 articles that were completely irrelevant to the research topic were preliminarily excluded. After reading the full texts, 11 articles were removed, and 13 articles [24–36] were ultimately included in the systematic review. The document screening process is shown in Figure 1.

### 3.2. Basic information of participants included in the study

The 13 included studies [24–36] spanned the period from 2019 to 2025. The source countries and regions were as follows: 8 studies from the United States (61%) [24,25,27–31,35], 1 from Spain [26], 1 from Australia [33], 1 from Japan [36], 1 from Taiwan [32], and 1 from India [34]. A total of 466 participants were included, comprising 396 males (85%) and 70 females (15%). Three studies (23%) focused on elite-level esports players [24,26,32], while ten studies (77%) focused on amateur esports players [25,27–31,33–36]. Eleven studies [24,25,27–30,32–36] reported participants' height, weight, and body composition indices. Four studies [25,27,29,30] reported participants' resting heart rate, systolic blood pressure, and diastolic blood pressure. Detailed information on the participants included in the studies is presented in Table 2. All 13 studies were RCTs [24–36], with 5 studies [28–31,33] being three-arm RCTs and 8 studies [24–27,32,34–36] being two-arm RCTs. Three studies [25,30,34] were randomised controlled studies, while 10 studies [24,26–29,31–33,35,36] were randomised controlled crossover trials with washout periods ranging from 2 to 14 days.

The supplement groups in 13 studies [24–36] employed 18 different supplement protocols. The control groups in 12 studies received placebos, while the control group in 1 study received purified water. The nutritional supplements administered in the 4 supplementation protocols included inositol-enhanced arginine silicate [25,27], mangiferin [27], and oral gamma-aminobutyric acid [36]. Fourteen supplementation protocols contained caffeine [24,26,28–33,35], and seven were pure caffeine intake [26,28,29,31–33], with doses of 1 mg/kg, 3 mg/kg, and 270 mg. The remaining 7 supplementation protocols [24,28–31,35] involved the combined intake of caffeine and other nutritional supplements, with caffeine supplementation doses ranging from 40 to 270 mg. Other nutrients included high/low doses of microalgae extracts [30], L-theanine [24], Caffeine + Dynamine + TeaCrine (CDT) [28,31], chlorogenic acid antioxidant [29], and multivitamins [35], among other nutrients, in capsule and energy drink forms. The basic information of study participants and supplementary protocols are detailed in Table 2. Outcomes and findings are presented in Tables 3a, 3b, 3c, 3d (a: cognitive; b: mood and psychological; c: gaming performance; d: adverse events).

**Table 3a.** Cognitive performance outcomes.

Study	Cognitive performance	Main conclusions
Thomas et al. [24]	EFT (Reload^TM^ : NS); Go/No-go test (Reload^TM^ : NS); *N*-back test (Reload^TM^ : NS)	No significant enhancement in cognitive/physical performance in elite players, except for working memory within the group.
Tartar et al. ^[25]^	TMT -B: Error rate (ASI + I ↓*); Other domains: NS	Acute and sustained improvements executive function.
Sowinski et al. [27]	Sternberg Task Test: 4 letter absence RT (ASI + I ↓*); 6 letter presentation RT (ASI + I ↓*); 6 letter absence RT (ASI + I ↓*)/APRT (ASI + I ↓*); AART (ASI + I ↓*); 4 letter absence accuracy(ASI + I ↑*); Average absence accuracy (ASI + I ↑*); Go/No-go test: accuracy (ASI + I ↑*); Other domains: NS	ASI + I can enhance reaction speed, working memory, and executive function in esports players undertaking high-cognitive-load tasks.
Tartar et al. [28]	Feanker task(CDT ↑*) (CAF ↑*); PVT RT (CDT ↓*); Other domains: NS	The CDT combination demonstrated superior performance to caffeine or placebo alone in inhibiting control, attention, and reaction time.
Bloomer et al. [29]	AXE- CPT accuracy (AmaTea®: NS/CAF: NS); AXE- CPT reaction time (AmaTea®: NS/CAF: NS); DSST(AmaTea®: NS/CAF: NS); Go/No-Go(AmaTea®: NS/CAF: NS)	No significant gaming/cognitive effects.
Leonard et al. [30]	BCST: NS; Go/No-go test: NS; Sternberg Task Test: NS; PVT: NS	High-dose PT combined with guarana can improve reaction time, executive function, attention, and working memory both in the short and long term.
Evans et al. [31]	*α* band frequency(CDT: ↓*); *θ* activity(CDT: ↑*)	The CTD combination enhances cognitive flexibility and executive control by modulating brain waves (reducing alpha and increasing theta).
Wu et al. [32]	Stroop RT; Congruent (CAF ↓*); Incongruent (CAF ↓*); Visual search RT (CAF ↓*)	Ingestion of 3 mg/kg caffeine significantly shortens reaction times and enhances information processing speed during high-cognitive-load tasks (such as multi-item visual searches) and the unconflicted Stroop task.
Rogers et al. [33]	PVT (CAF1 ↑*/CAF3 ↑*); Reactive tracking-accuracy (CAF1 ↑*/CAF3 ↑*)	Caffeine significantly enhances psychomotor alertness (reaction time), and even low doses (1 mg/kg) are sufficient to produce a marked effect.
Jeyakodi et al. [34]	DSST (Stadice® ↓*); Colour Trails Test 1 (Stadice® ↑*); DVT - errors (Stadice® ↓*); Stroop test (Stadice® ↓*); AVLT -Immediate (Stadice® ↑*); AVLT -Delayed (Stadice® ↑*)	Stadice® demonstrates significant improvements across multiple key cognitive domains, including mental processing speed, attention, response inhibition, verbal learning, and memory, with effects markedly superior to placebo.
Schwager et al. [35]	Cognitive flexibility (C4S ↑*); Executive function (C4S ↑*); Sustained attention (C4S ↑*); Motor speed (C4S ↑*); Psychomotor speed (C4S ↑*); Working memory (C4S ↑*)	C4S energy drinks deliver a broad and pronounced enhancement of higher-order cognitive functions, with particularly marked effects on cognitive flexibility, executive function, and sustained attention.

Abbreviation: EFT: eriksen flanker test, NS: not significant, TMT: trail making test, ASI+I: arginine silicate containing inositol, ↑: increase, ↓: decrease, *: significant difference (vs. control group), RT: reaction Time, APRT: average presentation reaction time, AART: Average absence reaction time, CDT: caffeine + dynamine + teacrine, CAF: caffeine, AXE - CPT: AXE-continuous Performance Task, DSST: digit symbol substitution test, BCST: berg-wisconsin card sorting test, PT: phaeodactylum tricornutum, PVT: psychomotor vigilance test, DVT: digit vigilance test, AVLT: auditory verbal learning test.

**Table 3b.** Mood and psychological outcomes.

Study	Mood and psychological	Main conclusions
Tartar et al. [25]	Vigour day 1 (ASI + I ↑*); Anger day 7 (ASI + I ↓*); Other domains: NS	ASI + I can enhance emotional states (such as increasing vigour and reducing anger).
Tartar et al. [28]	Alertness (CDT ↑*)	The CDT combination demonstrated superior tolerability in improving mood states, thereby avoiding the negative emotional effects associated with caffeine.
Bloomer et al. [29]	Vigour(AmaTea® ↑*); Fatigue(AmaTea® ↓*)	AmaTea demonstrates greater efficacy in enhancing emotional states, specifically vigour and fatigue.
Leonard et al. [30]	Vigour (HD ↑*); Other domains: NS	Supplements help maintain vitality and alleviate negative emotions.
Evans et al. [31]	POMS (CDT: ↓*); Alertness (CDT ↑*/CAF ↑*)	CTD improved overall mood but also increased feelings of anxiety; alertness was markedly elevated.
Rogers et al. [33]	Alertness (CAF1 ↑*/CAF3 ↑*); Tiredness:CAF1V↓*/CAF3 ↓*)	Caffeine enhances alertness, reduces feelings of fatigue, and improves subjective well-being.
Schwager et al. [35]	FI (C4S ↑*); VA (C4S ↑*); Friendliness (C4S ↑*); TMD (C4S ↓*)	C4S has demonstrated clear efficacy in boosting energy levels, reducing fatigue, and enhancing sociability, thereby significantly improving overall emotional well-being.
Hara et al. [36]	CB (GABA^®^ ↓*); FI (GABA^®^ ↓*); Other dimensions: NS	GABA intake can improve mental state (reducing confusion and fatigue).

Abbreviation: ↑: increase, ↓: decrease, *: significant difference (vs. control group), NS: not significant, ASI+I: arginine silicate containing inositol, CAF: caffeine, CDT: caffeine + dynamine + teacrine, HD: high dose, POMS: profile of mood states, FI: fatigue inertia, VA: vigour-activity, TMD: total mood disturbance, CB: confusion–bewilderment, GABA: γ-aminobutyric acid.

**Table 3c.** Gaming performance outcomes.

Study	Gaming performance	Main conclusions
Sainz et al. [26]	Simple reaction time (CAF ↓*); Hit reaction time (CAF↓*); Hit accuracy(CAF ↑*)	Caffeine reduces reaction time and improves accuracy in professional gamers.
Bloomer et al. [29]	Kill/Match: (AmaTea®: NS/CAF: NS); Other(AmaTea®: NS/CAF: NS)	No intervention was found to have a statistically significant effect on gaming performance, though AmaTea demonstrated a positive trend towards improvement.
Evans et al. [31]	Standard mode: Number of hits(CDT ↑*); Speed mode: Number of hits(CDT ↑*/CAF ↑*); Total kills (CDT ↑*); Precision mode: Kills/second:(CDT ↑*)	CTD outperforms either caffeine alone or a placebo in terms of speed, accuracy, and reaction time, particularly in maintaining speed without compromising accuracy.
Wu et al. [32]	Kill ratio (CAF ↑*); Kill accuracy (CAF ↑*); Average time to target(CAF ↓*)	Caffeine significantly enhances kill ratios, accuracy rates, and aiming speed in shooting games.
Rogers et al. [33]	Static clicking: Score (CAF1 ↑*/CAF3 ↑*); Hit rate (CAF1 ↑*/CAF3 ↑*); Accuracy: NS; Shoot fired: NS	Caffeine enhanced performance in both static click and reaction tracking tasks without compromising accuracy.
Schwager et al. [35]	Tetris(C4S ↑*); Other game: NS	C4S can effectively enhance performance in games demanding high cognitive effort and relying on visual-spatial working memory, such as Tetris.
Hara et al. [36]	Total score (GABA^®^ ↑*); Background processing (GABA^®^↑*); Mechanics tended: NS; Map awareness: NS	GABA intake enhances gaming performance (improving overall scores and background processing capabilities).

Abbreviation: ↑: increase, ↓: decrease, *: significant difference (vs. control group), NS: not significant, CAF: caffeine, CDT: caffeine + dynamine + teacrine, GABA: γ-aminobutyric acid.

**Table 3d.** Side effects and adverse events.

Study	Side effects and adverse events	Main conclusions
Tartar et al. [25]	Rare, no groups differences.	The supplement demonstrated good safety, with no significant side effects observed.
Sowinski et al. [27]	Mild, no groups differences.	Indicates that ASI + I exhibits good safety following acute administration.
Tartar et al. [28]	Anxiety: (CAF ↑*); Headache: (CDT ↓* VS CAF); Cortisol: ↑ (CDT ↑*)	The CDT combination demonstrated favourable safety profiles, with no significant adverse reactions observed.
Bloomer et al. [29]	Pain (*n* = 1), Syncope (*n* = 2); Jittery (CAF ↑*); SBP: ↑ (AmaTea® and CAF); Cortisol: ↑(AmaTea® and CAF)	The safety profile is favourable, with reported adverse events unrelated to the supplement itself.
Leonard et al. [30]	Mild, no groups differences.	The supplement was well tolerated over a 30-day period, with no significant side effects or impact on sleep.
Evans et al. [31]	Anxiety (CDT ↑*/CAF↑*); Cortisol (CDT ↑*/CAF↑*)	CTD is well tolerated with no serious side effects, though it may cause mild anxiety.
Schwager et al. [35]	The primary side effects include mild gastrointestinal discomfort and isolated cases of palpitations or itching. No serious cardiovascular risks have been observed.	The safety and tolerability of C4S are good when consumed as a single, short-term dose.

Abbreviation: ↑: increase, ↓: decrease, *: significant difference (vs. control group), NS: not significant, CAF: caffeine, CDT: caffeine+ dynamine + teacrine.

### 3.3. Methodological quality and assessment of risk of bias

The methodological quality of the 13 studies included in the systematic review [24–36] was evaluated using the PEDro scale, with the results presented in Table 4. The methodological quality scores ranged from 6 to 10 points, with an average score of 7.36 points. One study [26] demonstrated high methodological quality, scoring 10 points. The remaining 12 studies [24,25,27–36] exhibited good methodological quality, with 4 studies scoring 6 points, 4 studies scoring 7 points, and 4 studies scoring 8 points. Therefore, the conclusions drawn from these studies should be interpreted with caution. All studies reported random allocation, adequate follow-up, between-group statistical comparisons, point estimates, and variability [24–36]. Two studies [26,36] reported concealed allocation, 11 studies [24–31,33–35] reported baseline comparability, 12 studies [24–32,34–36] reported blinding of subjects, 7 studies [24–26,33–36] reported blinding of therapists, 6 studies [26,28,32–34,36] reported blinding of assessors, and 4 studies [24,26,32,35] reported intention-to-treat analysis.

**Table 4.** PEDro scores of studies included in the systematic review.

Study	I1	I2	I3	I4	I5	I6	I7	I8	I9	I10	I11	Total score
Thomas et al. [24]	1	1	0	1	1	1	0	1	1	1	1	8
Tartar et al. [25]	1	1	0	1	1	1	0	1	0	1	1	7
Sainz et al. [26]	1	1	1	1	1	1	1	1	1	1	1	10
Sowinski et al. [27]	1	1	0	1	1	0	0	1	0	1	1	6
Tartar et al. [28]	1	1	0	1	1	0	1	1	0	1	1	7
Bloomer et al. [29]	1	1	0	1	1	0	0	1	0	1	1	6
Leonard et al. [30]	1	1	0	1	1	0	0	1	0	1	1	6
Evans et al. [31]	1	1	0	1	1	0	0	1	0	1	1	6
Wu et al. [32]	1	1	0	0	1	0	1	1	1	1	1	7
Rogers et al. [33]	1	1	0	1	0	1	1	1	0	1	1	7
Jeyakodi et al. [34]	1	1	0	1	1	1	1	1	0	1	1	8
Schwager et al. [35]	1	1	0	1	1	1	0	1	1	1	1	8
Hara et al. [36]	1	1	1	0	1	1	1	1	0	1	1	8

Note: I: Item; I1. eligibility criteria specified; I2. random allocation; I3. concealed allocation; I4. baseline comparability; I5. blinding of subjects; I6. blinding of therapists; I7. blinding of assessors; I8. adequate follow-up; I9. intention-to-treat analysis; I10. between-group statistical comparisons; I11. point estimates and variability. 1 means eligible; 0 means ineligible. Item 1 is not included in the total score calculation.

Four studies [24,26,27,36] explicitly reported details of random sequence generation methods, indicating low risk; nine studies [25,28–35] did not report specific information on generation methods, raising some concerns. In the assessment of bias due to deviations from intended interventions, nine studies [24–31,36] were at low risk, while four studies [32–35] raised some concerns. For bias due to missing outcome data, 9 studies [24,26–28,32–36] were at low risk, and 4 studies [25,29–31] had some concerns. All studies were at low risk for bias in the measurement of the outcome and bias in the selection of the reported result. Overall, 5 studies [24,26–29,36] were at low risk for bias, and 8 studies [25,29–35] had some concerns. For specific information, see Table 5.

**Table 5.** Information on the risk of bias in studies included in the systematic review.

Study	Randomisation Process Bias	Bias due to deviations from intended interventions	Bias due to missing outcome data	Bias in measurement of the outcome	Bias in selection of the reported result	Overall bias
Thomas et al. [24]	Low risk	Low risk	Low risk	Low risk	Low risk	Low risk
Tartar et al. [25]	Some concerns	Low risk	Some concerns	Low risk	Low risk	Some concerns
Sainz et al. [26]	Low risk	Low risk	Low risk	Low risk	Low risk	Low risk
Sowinski et. al [27]	Low risk	Low risk	Low risk	Low risk	Low risk	Low risk
Tartar et al. [28]	Some concerns	Low risk	Low risk	Low risk	Low risk	Low risk
Bloomer et. al [29]	Some concerns	Low risk	Some concerns	Low risk	Low risk	Some concerns
Leonard et. al [30]	Some concerns	Low risk	Some concerns	Low risk	Low risk	Some concerns
Evans et al. [31]	Some concerns	Low risk	Some concerns	Low risk	Low risk	Some concerns
Wu et al. [32]	Some concerns	Some concerns	Low risk	Low risk	Low risk	Some concerns
Rogers et al. [33]	Some concerns	Some concerns	Low risk	Low risk	Low risk	Some concerns
Jeyakodi et. al [34]	Some concerns	Some concerns	Low risk	Low risk	Low risk	Some concerns
Schwager et al. [35]	Some concerns	Some concerns	Low risk	Low risk	Low risk	Some concerns
Hara et al. [36]	Low risk	Low risk	Low risk	Low risk	Low risk	Low risk

### 3.4. The effects of different nutritional supplements on esports players

#### 3.4.1. Caffeine

Regarding cognitive function performance, Tartar et al [28]. reported that caffeine significantly improved performance on the Flanker task. Wu et al [32].reported that caffeine significantly improved reaction time and visual search time on the Stroop test. Rogers et al [33]. reported that caffeine significantly improved reaction time on the psychomotor vigilance test (PVT) task.

In terms of gaming performance, Sainz et al [26]. and Wu et al [32]. both reported that caffeine significantly improved simple reaction times, hit accuracy, and kill ratios in games. Rogers et al [33]. further confirmed that caffeine yielded superior performance in static click and reaction tracking tasks. Evans et al [31]. reported that the caffeine group achieved higher hit counts than the placebo group in speed mode.

Regarding psychological mood, Rogers et al [33]. reported that caffeine significantly enhances subjective alertness and reduces tiredness. Evans et al [31]. also documented a significant increase in alertness among the caffeine group.

#### 3.4.2. Caffeine-containing mixture

In terms of cognition, Thomas et al [24]. reported that energy drinks containing caffeine mixtures improved working memory only within groups, with no significant differences in cognitive function observed between groups compared to placebo. Tartar et al [28]. reported significantly enhanced Flanker attention, significantly reduced PVT reaction times, and significantly increased Delta and Theta waves in the CDT supplement group. Bloomer et al [29]. reported no significant differences in cognitive function between the supplement and placebo groups. Evans et al [31]. reported enhanced cognitive flexibility and alertness (significantly reduced alpha wave power) in the CDT supplement group, along with improved executive control and decision-making abilities (significantly increased theta wave power). Schwager et al [35]. documented significant improvements in the C4S energy drink supplement group (containing caffeine) across cognitive flexibility, executive function, sustained attention, motor speed, psychomotor speed, and working memory.

In terms of gaming performance, Bloomer et al [29]. found that the AmaTea group achieved 21% more kills per match than the placebo group and 12% more than the caffeine group, though these differences were not statistically significant. Evans et al [31]. observed that the CDT supplement group significantly outperformed others in standard mode with more hits on targets and total shots fired, in speed mode with more hits on targets and total kills, and in precision mode with significantly higher kills per second. Schwager et al [35]. observed significantly higher Tetris scores in the C4S energy drink group.

Regarding psychological mood, Tartar et al [28]. reported higher alertness in the CDT supplement group. Bloomer et al. found significantly increased vitality and reduced fatigue in the AmaTea group, with no between-group differences in other dimensions. Evans et al [31]. observed significantly reduced overall emotional distress and significantly increased alertness in the CDT supplement group. Schwager et al. ^[35]^ observed significant improvements in the C4S energy drink group across total emotional distress, fatigue-lethargy, vitality-activity, and agreeableness.

#### 3.4.3. Inositol-enhanced arginine silicate

In terms of cognitive performance, Tartar et al [25]. reported that the ASI group demonstrated superior improvement in trail making test part B (TMT-B) error rates compared to the placebo group, while improvements in other cognitive dimensions showed no significant difference from the placebo group. Sowinski et al [27]. reported that the ASI group exhibited greater improvement than the placebo group in overall performance on the Sternberg Task Test and in accuracy on the Go/No-go test.

Regarding psychological mood, Tartar et al [25]. reported that the ASI group scored significantly higher than the placebo group on the “vitality” subscale and significantly lower on the “anger” subscale.

#### 3.4.4. Other nutritional supplements

In terms of cognition, Leonard et al [30]. found no significant difference between the PT + guarana group and the placebo group in cognitive function improvement. However, Jeyakodi et al [34]. observed that the Stadice group demonstrated superior improvements compared to placebo in mental speed, attention focus, sustained attention, response inhibition, and verbal learning memory.

Regarding psychological mood, Hara et al [36]. reported significant reductions in confusion-disorientation and fatigue-lethargy in the GABA group. No significant differences were observed in other dimensions. Leonard et al. found significantly increased vitality in the supplement group compared to placebo.

Regarding game performance, Hara et al [36]. reported significant improvements in total game scores and background processing scores in the GABA group. Leonard et al. found no significant difference in game performance improvement between the supplement group and the placebo group.

### 3.5. Side effects or adverse events

None of the studies reported serious adverse events. Six studies [24,26,32–34,36] made no mention of side effects or minor adverse events caused by nutritional supplements, while seven other studies [25,27–31,35] did. Specifically:

Tartar [25] and Sowinski et al [27]. noted that ASI + I may induce headaches, dizziness, palpitations, and tachycardia, though no significant differences were observed compared to the placebo group;

Tartar et al [33]. found caffeine significantly increased anxiety and headaches, with markedly higher cortisol levels in the CDT group;

Bloomer et al [29]. observed greater tension in the caffeine group, along with significant increases in systolic blood pressure and cortisol levels, plus one case of puncture site inflammation and two cases of transient syncope;

Leonard et al [30]. noted PT + guarana may induce dizziness and palpitations, though incidence and severity showed no intergroup differences;

Evans et al [31]. confirmed significantly elevated anxiety levels and cortisol concentrations in both CDT and caffeine groups;

Schwager et al [35]. documented C4S energy drinks primarily causing mild gastrointestinal discomfort, with occasional palpitations or itching, and no serious cardiovascular risks observed.

## 4. Discussion

### 4.1. Summary of evidence

This systematic review included 13 RCTs [24–36] to assess the effects of nutritional supplements on cognitive performance, gaming performance, and psychological mood in esports athletes. The supplementation groups in the 13 studies [24–36] used 18 different nutritional supplementation protocols, with 14 of these protocols containing caffeine. A previous scoping review assessed the role of dietary supplements in the cognitive performance of competitive gamers [10]. Unlike prior studies, our research selected more direct evidence (RCTs) as inclusion criteria to evaluate the effects of different nutritional supplements versus placebo on competitive gamers. We also updated recent randomised controlled trials involving novel supplements such as *γ*-aminobutyric acid and mangiferin. Beyond cognitive function, our findings report the effects of nutritional supplements on players' gaming performance, psychological and emotional states, and the occurrence of adverse events.

The effects of pure caffeine on esports players' cognitive performance primarily manifest in reduced reaction times for task completion; its enhancement of gaming performance is mainly reflected in improved shooting accuracy and kill ratios; its impact on psychological mood is primarily evident in heightened alertness and reduced fatigue. The effects of caffeine mixtures on esports players' cognitive performance extend across multiple dimensions, encompassing improvements in cognitive flexibility, attention, and executive function; its impact on gaming performance also manifests as improved shooting accuracy and kill ratios in FPS games; psychological mood effects extend beyond alertness and fatigue reduction to include overall emotional distress and enhanced vitality. ASI + I influences esports players' cognitive function by increasing task accuracy in testing; psychological improvements primarily involve heightened vitality. None of the included studies reported serious adverse events. However, some studies noted potential side effects of nutritional supplements: caffeine may elevate cortisol levels in players and induce symptoms like anxiety, tension, and headaches. Other supplements caused minor symptoms, but these showed no significant difference compared to the control group.

However, these findings provide only preliminary evidence regarding the effects of nutritional supplements on esports players. This is because the findings are derived from randomised crossover/controlled trials with small sample sizes. Furthermore, eight of the thirteen studies included were judged to have an overall risk of bias that was “of some concern.” Finally, the heterogeneity between intervention protocols and outcome measures limits the feasibility of further meta-analyses.

### 4.2. Interpretation of results

Among the 18 nutritional supplementation regimens we examined, 14 were found to be related to caffeine. As an active substance that effectively serves as a dietary supplement for bodily functions, caffeine is highly favoured by athletes of all kinds [37,38]. Its chemical structure is similar to adenosine, and it competitively blocks adenosine receptors [39], counteracting the inhibitory effects of adenosine, enhancing neuronal activity, reducing fatigue, and improving alertness [40]. The effects of caffeine on cognition have been extensively reviewed, such as improving attention and reaction time during exercise [41], counteracting sleep deprivation [42], and reducing the risk of cognitive impairment [43]. The three studies included in this review [26,32,33] used a caffeine dose of 3 mg/kg (weight). The results showed that caffeine significantly improved cognitive function and gaming performance in esports players compared to a placebo [26,32,33]. In terms of psychological mood improvements, caffeine supplements demonstrated clear efficacy, particularly in enhancing vitality and reducing negative emotions [29–31,35]. This aligns with the findings of previous studies [44,45]. Previous studies have suggested that caffeine intake within the range of 0.5 to 4 mg·kg^–1^ can enhance basic aspects of cognitive function, such as reaction time, attention, and alertness [46]. In Rogers et al.'s study [33], although there was no statistically significant difference between 1 mg/kg and 3 mg/kg in improving shooting scores and PVT reaction time, the higher dose showed advantages in reaction tracking accuracy and PVT reaction time. The caffeine intake range in current studies spans 40–270 mg, though this falls within the recommended range. However, some studies incorporated other nutrients, and only three studies standardised pure caffeine intake, making cross-study comparisons challenging. Therefore, future research should employ standardised cognitive performance and gaming performance assessment tools whilst administering weight-adjusted caffeine doses (e.g. 3–6 milligrams per kilogram). This will aid in clarifying the dose-response relationship between cognitive and psychological benefits in esports athletes.

ASI + I, as an active nutritional component, can increase levels of arginine, silicon, and nitric oxide in the blood, thereby improving cerebral blood flow and neural transmission [47]. Inositol, as a stabiliser, enhances the bioavailability of arginine and participates in neurotransmitter regulation [48]. The two studies included in this review [25,27] used a dosage of 1500 mg of arginine silicate + 100 mg of inositol. The results showed that ASI + I effectively improved executive function in esports players, including cognitive flexibility [25], working memory [27], and inhibitory control [27]. In terms of psychological mood, vitality scores improved significantly, and anger scores decreased more than in the placebo group [25]. Previous studies have shown that short-term supplementation with ASI can significantly improve cognitive flexibility and memory in healthy men [49,50]; enhance self-perceived energy levels without cardiovascular side effects [51]; and prevent post-exercise cognitive decline [52].

GABA (gamma-aminobutyric acid) is an amino acid that influences psychological mood. As the primary inhibitory neurotransmitter in the central nervous system, it is naturally present in fruits, vegetables, and grains, with high safety [53]. By activating the parasympathetic nervous system, it increases brain waves and reduces salivary cortisol. Parasympathetic dominance is associated with shorter reaction times and better cognitive performance [54]. One study [36] included in this review found that after ingesting 200 mg of GABA, players experienced a significant reduction in negative emotions such as fatigue and confusion, and their overall gaming performance scores and background processing abilities were significantly enhanced. However, the study sample consisted of a small group of college esports players with moderate experience, so future research designs should consider larger sample sizes and more diverse groups, such as professional esports players.

Among the 13 studies included in this research, only 70 female participants (15%) were identified, nearly all of whom were amateurs. Elite players numbered only 33 cases (7%), all of whom were male. This stark gender disparity stems from women's severe under representation in esports. Within the esports community, elite female players face multiple professional barriers. The industry is rife with harassment, fostering a highly hostile cultural environment for women. Women require extraordinary perseverance to sustain their professional careers [55,56]. Among elite players, participation in relevant trials may potentially conflict with players' regular training schedules and club management policies. This could represent a potential obstacle to achieving an adequate sample size of elite players.

### 4.3. Clinical practice implications and future research directions

This systematic review examined the effects of nutritional supplements on cognitive performance, psychological mood, and gaming performance among esports players. We found that nutritional supplements may offer potential benefits for these aspects of performance among esports players. However, we also identified several limitations in the current research designs. 1) Small sample sizes: Most studies focused on casual players, with limited samples of elite players; there was significant gender bias, with fewer female players included. 2) High heterogeneity in supplementation protocols and measurement results: There is a lack of consistency in the diversity and dosage of supplementation substances; there is also high heterogeneity in the methods used to measure cognitive/gaming performance. 3) Only some studies preregistered their protocols prior to publication and reported adverse events after the study. Therefore, future research designs should prioritise the inclusion of professional players and female populations in participant selection, such as maintaining balanced gender ratios or conducting studies specifically targeting women. Measurement methods should standardise cognitive/gaming performance metrics (e.g. reaction time, kill-to-death ratio). Pre-registration of experimental protocols and reporting of adverse events should also be emphasised in forthcoming studies. Beyond this, future research designs should also prioritise intelligent data collection methods, such as integrating wearable devices with sensors. This approach enables more effective monitoring of esports players' real-time physiological states and overcomes the limitations of traditional cognitive testing and subjective questionnaire data collection [57].

### 4.4. Limitations

This study has several limitations: 1. We included only four English-language electronic databases, omitting studies published in other languages within non-English databases—particularly in regions like Southeast Asia, where esports are more prevalent—which may introduce language bias. 2. No formal assessment of publication bias was conducted (funnel plot, Egger's test). 3. Inability to conduct meta-analysis: Significant heterogeneity in supplementation regimens and outcome measures across the included studies precluded a formal meta-analysis.

## 5. Conclusion

Due to methodological limitations and the heterogeneity of included studies, our research provides only preliminary evidence of nutritional supplements' benefits for esports players. These benefits encompass enhanced cognitive function manifested in improved attention and executive function, both closely linked to gaming performance; psychological and emotional improvements reflected in increased vitality and reduced negative emotions; and elevated gaming performance demonstrated through shorter shooting reaction times, improved accuracy, and higher game scores. However, when interpreting these findings, two aspects warrant attention: the sample size was relatively small, and participants were predominantly amateur athletes; coffee-based supplements constituted the bulk of nutritional supplements, whilst evidence for other novel nutritional supplements remains extremely limited. Future research designs should prioritise standardisation of supplementation protocols and measurement procedures.

## Supplementary Material

Supplementary material Supplementary Information

## Data Availability

For further information, please contact the corresponding author.
